# Pembrolizumab response in stage IV luminal-type breast cancer with high microsatellite instability: a case report

**DOI:** 10.1186/s13256-024-04522-2

**Published:** 2024-05-01

**Authors:** Keiko Inakami, Noriko Fujita, Chikage Iguchi, Yukie Enomoto, Junya Minohata, Atsushi Sata, Yoshimasa Miyagawa, Tetsu Yanagisawa, Tomokazu Saitoh, Takashi Nomura, Yuka Sawai, Keiko Takahara, Tsutomu Kasugai, Eiichi Shiba

**Affiliations:** 1 Department of Breast and Endocrine Surgery, Osaka Breast Clinic, 1-13-8 Ohiraki Fukushima, Osaka, 553-0007 Japan; 2 Department of Radiology, Osaka Breast Clinic, Osaka, Japan; 3 Department of Radiotherapy, Osaka Breast Clinic, Osaka, Japan; 4 Department of Pathology, Osaka Breast Clinic, Osaka, Japan

**Keywords:** Breast cancer, Pembrolizumab, Microsatellite instability, Immune checkpoint inhibitor, Luminal type

## Abstract

**Background:**

Pembrolizumab (PEM), an immune checkpoint inhibitor (ICI), is often used for triple-negative breast cancer, but can also be used to treat solid tumors that exhibit high microsatellite instability (MSI-High). However, patients with breast cancer rarely have MSI-High, the use of PEM in such cases in clinical practice is uncertain due to lack of sufficient supporting data. Here, we report the case of a premenopausal woman in who received PEM for MSI-High luminal-type breast cancer.

**Case presentation:**

A 40-year-old premenopausal Asian woman was diagnosed with stage IIA (T2N0M0) breast cancer and had an Oncotype DX recurrence score of 38. After surgery, she received 4 courses of chemotherapy with docetaxel and cyclophosphamide. After 3 months of tamoxifen therapy, the patient complained of abdominal pain due to right iliac metastasis, and biopsy of the metastatic lesion showed of luminal type; she was sequentially treated with fulvestrant, a CDK4/6 inhibitor, and an anticancer drug (TS1), but over the next year, metastasis to the bone and para-aortic lymph nodes increased. Tumor was MSI-High; PEM was started, and after three courses, bone metastases were reduced, para-aortic lymph node metastases resolved, opioids were discontinued, and the patient returned to society; PEM was administered for 1 year with no worsening of bone metastases on imaging. Asymptomatic brain metastasis less than 1 cm was detected and gamma knife was performed. Six months after completion of PEM, the patient is working with no new lesions.

**Conclusion:**

We report a case of luminal-type breast cancer with bone metastases and MSI-High, which was treated with PEM and showed a rapid therapeutic response.

## Introduction

Immune checkpoint inhibitors (ICIs) have recently revitalized cancer therapy. Pembrolizumab (PEM) has been approved to treat advanced or recurrent solid tumors with high microsatellite instability (MSI), which are refractory to standard chemotherapy regimens [1, 2]. The KEYNOTE-158 trial showed that PEM produced promising responses in tumors with high-frequency microsatellite instability (MSI-High) [3]. Studies have reported that the prevalence of MSI-High in breast cancers is approximately 0.5–1.7% [4]. Therefore, the use of PEM in patients with MSI-High breast cancer is not common.

The overall survival is considered good for bone metastases in breast cancer alone, even at stage IV [5]; however, we report a case of luminal-type breast cancer with exacerbating bone metastases in the short term, in which PEM was successfully used early in the disease course.

## Case presentation

The patient was a 40-year-old premenopausal Asian woman with no known medical history. Her father had died of multiple sclerosis, and the patient had no other family history of malignant tumors. She had a preoperative thoracoabdominal Computed Tomography (CT) and abdominal echocardiography that revealed no lesions in other organs. The preoperative diagnosis was left upper lateral breast cancer T2N0M0 stage IIA, and left nipple-sparing mastectomy and sentinel node biopsy (0/5) were performed. The postoperative pathological diagnosis was invasive ductal carcinoma with a tumor diameter of 30 × 35 mm, no vascular invasion, histological grade II, hormone receptor (HR) + (estrogen receptor: 90% A-S: 3 + 5 = 8, progesterone receptor: 99% A-S: 3 + 5 = 8), Ki-67 80%, human epidermal growth factor receptor (HER)2 −, and *BRCA*: mutation-negative. The Oncotype Dx of the surgical specimen showed a high recurrence score of 38.

**Fig. 1:**
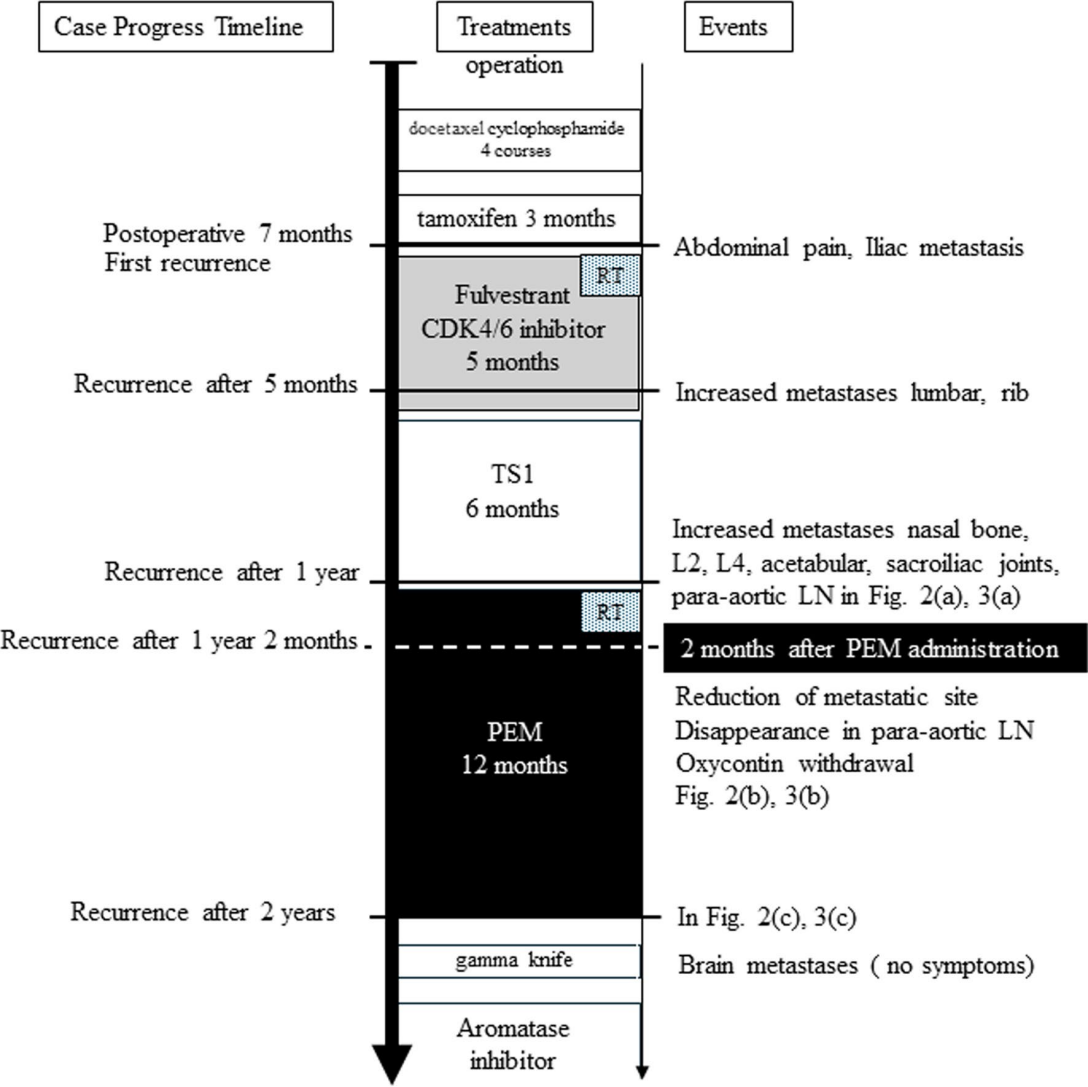
Time course of metastases and treatments, events

Her clinical course and treatments are summarized in Fig. 1. Postoperatively, after 3 months of docetaxel and cyclophosphamide therapy (4 courses) and 3 months of hormone therapy (tamoxifen), abdominal pain was observed. The cause of her abdominal pain is a 4-cm bone metastasis in the right iliac bone. The bone biopsy showed HR +, HER2 −, Ki-67 60%. Intensity-modulated radiation therapy (IMRT) was administered to the patient’s right iliac bone. Tamoxifen was discontinued, and a treatment using LH-RH agonist, fulvestrant, CDK4/6 inhibitor (Abemaciclib) and denosumab was initiated.

In the fifth month after bone metastases were known, metastasis was found in the lumbar spine and right 10th rib, and the treatment was deemed to be ineffective for progressive disease. Hormonal therapy was discontinued and replaced with the anticancer drug, Tegafur Gimeracil Oteracil Potassium (TS1) as it has no hair loss and is superior in quality of life.

**Fig. 2:**
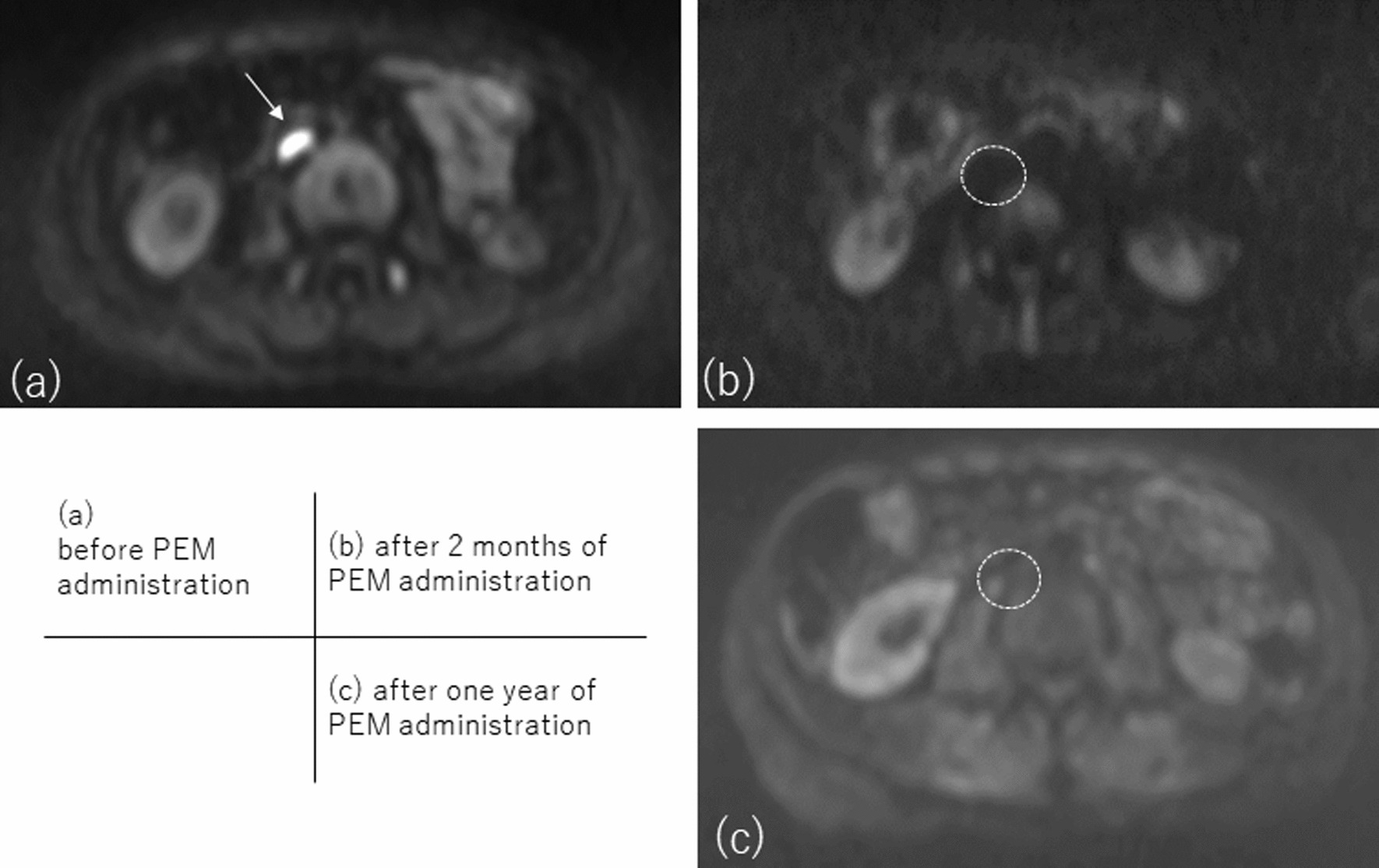
Progress of PEM treatment of para-aortic lymph node metastasis of breast cancer, using deffusion weighted MRI: **a** before PEM administration, the para-aortic lymph nodes were metastatic (arrow). **b** after 2 months of PEM administration, para-aortic lymph nodes in the circular area were disappeared. **c** After one year of PEM administration, the para-aortic lymph nodes in the circular area remain obliterated

**Fig. 3:**
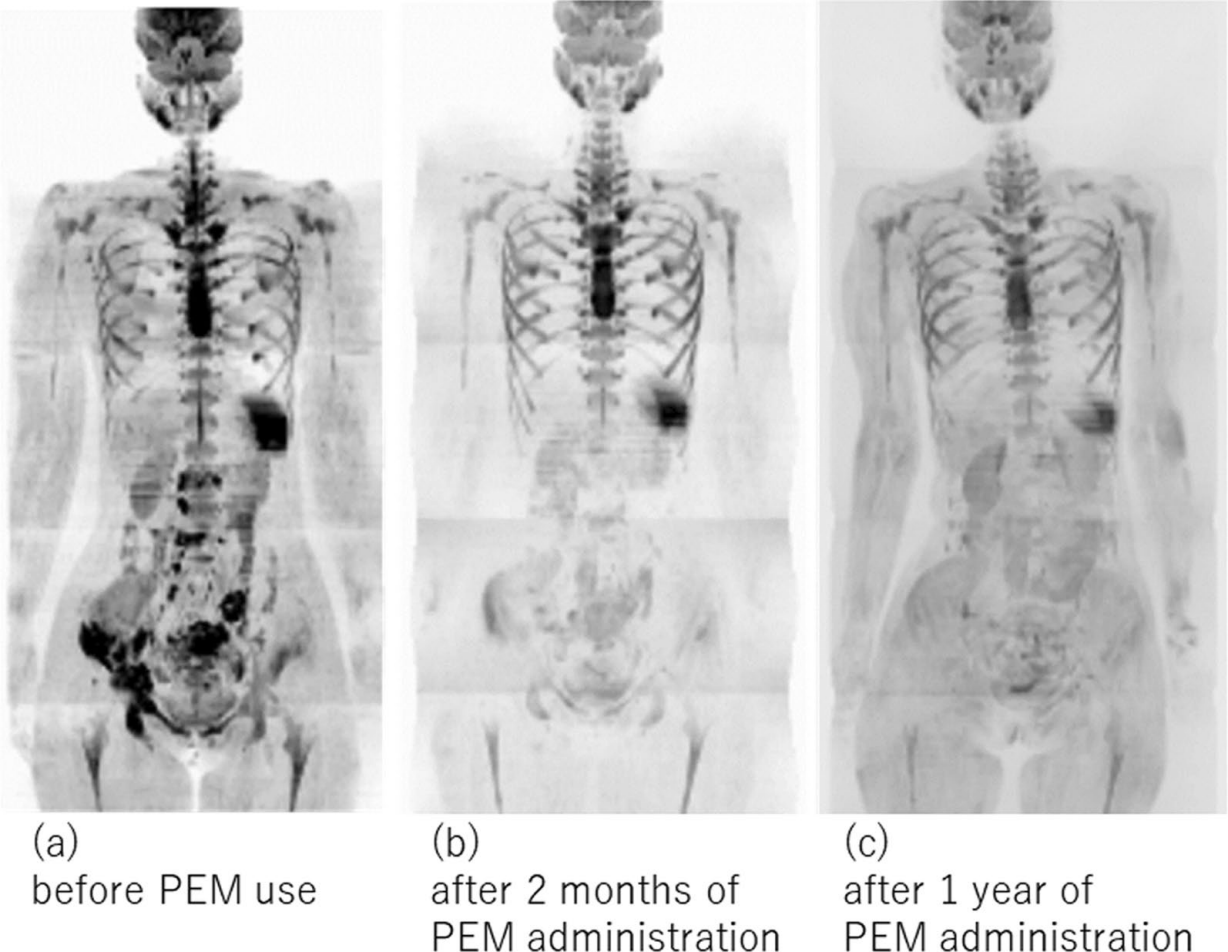
Pembrolizumab (PEM) treatment course in breast cancer with multiple metastasis photographed using diffusion-weighted whole-body imaging with background body signal suppression (DWIBS): **a** before PEM administration, showing metastases to the L2, L4 right, and left sacroiliac joints, right ilium, acetabulum, nasal bone, right iliac bone and the sacral tumor. **b** After 2 months of PEM administration, DWIBS revealed an overall increase in apparent diffusion coefficient values and shrinkage in tumor volume. **c** After one year of PEM administration, the signal of the metastatic site on diffusion-weighted imaging remains low

One year after bone metastases were known, Magnetic Resonance Imaging (MRI) showed new metastases to the L2, L4 right, and left sacroiliac joints, right ilium, acetabulum, and nasal bone. The right iliac bone undergoing radiation therapy enlarged; the sacral tumor enlarged, and she developed metastatic para-aortic lymph nodes in Figs. 2a, 3a. Blood tests showed that tumor markers (CEA, CA15-3) were normal, but serum lactate dehydrogenase was abnormally high at 838 IU/L.

The MSI-IVD Kit (FALCO, Kyoto, Japan) specifically detects MSI-High in tumor tissue using five markers (*BAT25, BAT26, NR21, NR24, MONO27*) with single nucleotide repeat sequences that are less susceptible to genetic diversity. If more than two of the five MSI markers are MSI-positive, the patient is considered MSI-High, and PEM is indicated in such cases. The patient’s breast cancer tissue was positive for all five markers.

She used PEM as the 3rd treatment after recurrence. Her pelvic region was treated with IMRT for 2 weeks, and PEM (200 mg) was administered every 3 weeks with zoledronic acid. She was admitted to due to fever and malaise, but was recovered within a few days. Two months after PEM administration (after three courses of PEM), Diffusion-weighted Whole body Imaging with Background Suppression (DWIBS) revealed an overall increase in apparent diffusion coefficient values and shrinkage in tumor volume (Figs. 2b, 3b). The para-aortic lymph nodes also disappeared, opioid use was discontinued, and LDH levels trended downward. She was taken off OxyContin and returned to work.

Her treatment with PEM continued for one year with no exacerbation of metastases, the signal of the metastatic site on diffusion-weighted imaging remains low (Figs. 2c, 3c). She was able to keep her job, though, due to loss of appetite, treatment was stopped. We suspected we had missed some major lesion and decided to investigate its cause. This was two years after the bone metastasis was discovered. CT and colonoscopy showed no evidence of malignancy in the abdominal cavity. Gastroscopy revealed pyloric stenosis, she was put on prednisone, appetite returned, and so the diagnosis of irAE due to PEM was made. She was not diagnosed with Lynch syndrome, but a head MRI was performed to rule out brain tumors, which can be seen in Lynch syndrome [6]. Head MRI showed 4 brain metastases up to 1 cm in size, and gamma knife was performed. Fortunately, she is symptom free and has returned to work while taking aromatase inhibitors after 8 months of withdrawal of PEM (One year and 8 months after the start of PEM).

## Discussion

Although PEM has been approved for use in patients with MSI-High metastatic disease and no satisfactory alternative treatment, only five breast cancer patients were included in the KEYNOTE-158 trial, which included the largest cohort of non-colorectal MSI-High cancer, and breast cancer-specific response rates were not demonstrated [3]. In this case PEM was used as early as the third line of treatment for recurrence and was successful; in Luminal type recurrence, hormonal therapy is the mainstay and MSI measurements are often not considered early on. A PubMed search for MSI-High of Luminal type breast cancer patients with metastases treated with PEM was conducted. Vidula *et al.* [7] had a 245 days response in 5th line PEM treatment but used PEM for only 29 days in 10th line treatment. Kawamata *et al.* [8] used PEM in 5th line treatment of recurrence and had a 12 months response. Although less reported, measuring MSI early in the course of known metastasis and using PEM early in the course of disease may be worth considering if hormonal therapy is not successful.

If PEM has been successful in metastatic recurrence and there is clear imaging improvement, as in this case, we are not sure how long to continue treatment with PEM. The reasons are that PEM is expensive and we do not know when irAE will occur. The patient has reintegrated into the community, but should continue to be monitored for the development of irAE by monitoring blood tests and symptoms. Also, genetic testing to rule out Lynch syndrome has not been done because she refused [6] and will be reiterated.

We observe her treatment effect by imaging evaluation such as CT, DWIBS, and so on. Klouch *et al.* [9] to monitor treatment effect, reported that when the triple-negative breast cancer patient of MSI-High with metastases was given PEM and plasma microsatellites were measured, they were reduced and the patient’s general condition improved. If plasma levels of microsatellite instability correlate with the need for PEM, then the decision to continue treatment with PEM would be simpler and less expensive. Further research is awaited.

## Conclusions

We have observed a positive therapeutic response after only three courses of PEM as the third-line treatment for recurrent luminal-type breast cancer with rapidly exacerbating bone metastases. She admitted to asymptomatic brain metastases but has returned to work. This case is an example of success with early introduction of PEM in MSI-High metastatic luminal-type breast cancer.

## Data Availability

The datasets generated during and/or analyzed during the current study are available from the corresponding author upon reasonable request.
